# Supplementary material for: Content-rich biological network constructed by mining PubMed abstracts
Source: BMC Bioinformatics. 2004 Oct 8;5:147. doi: 10.1186/1471-2105-5-147 (PMC528731; doi:10.1186/1471-2105-5-147)
Supplement: Additional File 2 — The original results of the above study (non-essential files are deleted to keep the file size under the limit set by BMC bioinformatics). [file 1471-2105-5-147-S2.bz2 › chilibotAdditionalFile2/dip05/371ID10950949E6046/html/FGF2_FGFR1.html]

 


 **FGF2** and **FGFR1** 
  
Found 575 abstracts in PubMed, retrieved 05.  
 

 What does Google say? 
 PDF only 
| .edu only 

---

**Interactive relationship** (e.g. stimulation, inhibition, etc)

**Stimulatory relationship**- In situ hybridization analyses of TRalpha knockout mice, which have impaired ossification and skeletal mineralization, revealed reduced  **FGFR1**  mRNA expression in osteoblasts and osteocytes, whilst T3 failed to stimulate  **FGFR1**  mRNA or enhance  **FGF2**  activated MAPK signaling in TRalpha null osteoblasts.  Ref: 12805413 Mol Endocrinol< MedlineTA>Mol Endocrinol,
**Neutral relationship**

**Non-interactive relationship** (e.g. studied together, co-existance, homology, etc.)

- We also measured components of the FGF 2  [ **FGF2** ]  ternary signaling complex, fibroblast growth factor receptor 1  [ **FGFR1** ]  FGFR 1  [ **FGFR1** ]  and syndecan 4.  Ref: 12832283 Am J Physiol Lung Cell Mol Physiol< MedlineTA>Am J Physiol Lung Cell Mol Physiol,
- Similarly, T3 enhanced  **FGF2**  activated autophosphorylation of  **FGFR1** , but did not modify  **FGF2**  induced phosphorylation of the docking protein FRS2.  Ref: 12805413 Mol Endocrinol< MedlineTA>Mol Endocrinol,
- These changes are associated with specific alterations in the regulation of FGF 2  [ **FGF2** ] , FGFR 1  [ **FGFR1** ]  and syndecan 4 in the airway epithelial mesenchymal trophic unit, which may be associated with the developmental problems of the lung associated with exposure to ozone.  Ref: 12832283 Am J Physiol Lung Cell Mol Physiol< MedlineTA>Am J Physiol Lung Cell Mol Physiol,
